# Beyond starving cancer: anti-angiogenic therapy

**DOI:** 10.1007/s10396-023-01310-1

**Published:** 2023-05-12

**Authors:** Kyoko Hida, Nako Maishi, Aya Matsuda, Li Yu

**Affiliations:** Vascular Biology and Molecular Pathology, Faculty and Graduate School of Dental Medicine, Hokkaido University, N13 W7 Kita-Ku, Sapporo, 060-8586 Japan

**Keywords:** Tumor blood vessel, Tumor endothelial cell, Tumor microenvironment

## Abstract

Tumor blood vessels contribute to cancer progression by supplying nutrients and oxygen to the tumor, removing waste products, and providing a pathway to distant organs. Current angiogenesis inhibitors primarily target molecules in the vascular endothelial growth factor (VEGF) signaling pathway, inhibiting cancer growth and metastasis by preventing the formation of blood vessels that feed cancer. They also normalize vascular structural abnormalities caused by excess VEGF and improve reflux, resulting in increased drug delivery to cancer tissue and immune cell mobilization. As a result, by normalizing blood vessels, angiogenesis inhibitors have been shown to enhance the effects of chemotherapy and immunotherapy. We present findings on the characteristics of tumor vascular endothelial cells that angiogenesis inhibitors target.

## Tumor angiogenesis

Dr. Folkman was the first to propose the concept of cancer treatment by inhibiting angiogenesis [1]. As a surgeon, he noticed that tumor tissues were prone to bleeding and wondered if cancer caused angiogenesis. He believed that by inhibiting angiogenesis, he could control cancer. This marked the start of tumor angiogenesis inhibitor therapy [1]. Most anticancer drugs at the time targeted cancer cells, but his angiogenesis inhibitor therapy concept was novel in that it targeted tumor blood vessels. Initially, this concept was not widely accepted, but he and his colleagues began to identify angiogenic factors, such as vascular endothelial growth factor (VEGF) and basic fibroblast growth factor (bFGF), and demonstrated that angiogenic factors are required for cancer growth. Their efforts resulted in bevacizumab, the world’s first angiogenesis inhibitor, a humanized VEGF-neutralizing antibody [2] (described later). As a strategy to “feed (starve?) the army” of cancer, angiogenesis inhibitors have thus become one of the paradigm shifts in cancer therapy.

**Fig. 1:**
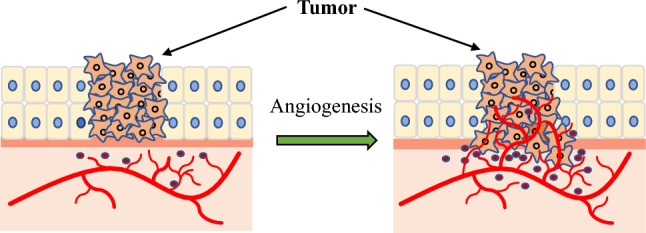
Cancer that has not invaded the basement membrane within a few millimeters (left). When the angiogenesis switch is turned on, cancer begins to grow and become invasive (right)

Cancers can survive at sizes as small as 1–2 mm, because they can obtain oxygen and nutrients through diffusion from their surrounding vessels. In a dormant state, such small tumors do not metastasize and are not life-threatening. Angiogenesis (angiogenic switch) occurs when cancer awakens from a dormant state and cancer cells begin to secrete angiogenic factors, such as VEGF, and cancer rapidly grows in size and malignancy [3] (Fig. 1).

**Fig. 2:**
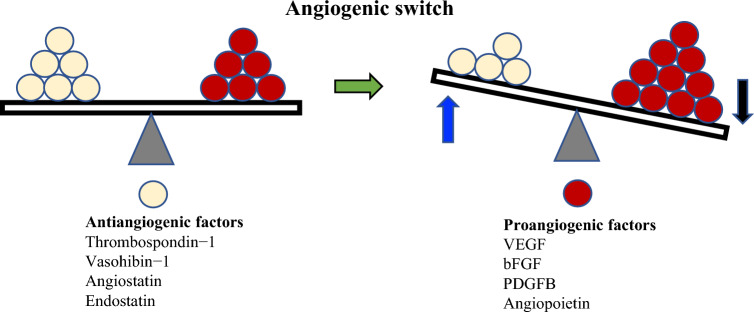
Angiogenesis is controlled by the balance of anti-angiogenic factors and pro-angiogenic factors. When pro-angiogenic factors become excessive, the angiogenic switch is turned on. In cancer tissue, the angiogenesis switch continues to be turned on, and angiogenesis is activated

The balance of multiple angiogenic factors and internal angiogenesis inhibitors regulates the angiogenic switch, and in cancer, an excess of angiogenic factors results in increased angiogenesis (Fig. 2).

The most prominent angiogenic factor is VEGF-A, whose gene expression is stimulated by hypoxia, cell proliferation signals, and tumor suppressor gene mutations. As a result, VEGF-A and its receptor, VEGF receptor (VEGFR), play critical roles in tumor angiogenesis and are important targets for cancer therapy. To induce angiogenesis, VEGF-A binds to VEGFR1 and VEGFR2 on endothelial cells and promotes their migration and proliferation [4]. VEGF-A also causes an increase in vascular permeability.

### Vascular abnormalities in tumors

**Fig. 3:**
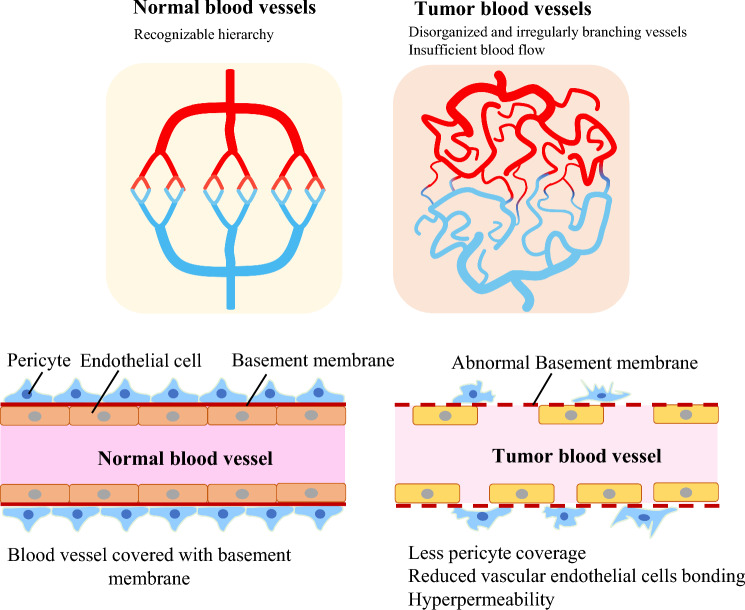
Tumor blood vessel abnormality. Tumor blood vessels (right) have an immature structure compared to normal blood vessels (left). As a result, the permeability of blood vessels increases, and blood flow is insufficient

The majority of current angiogenesis inhibitors block VEGF, its receptor VEGFR, and the signaling pathway. Rapidly growing cancers produce large amounts of angiogenic factors, and the balance of angiogenesis-promoting and angiogenesis-inhibiting factors tends to favor the promoting factors, resulting in rapid angiogenesis induction. Tumor blood vessels frequently have an immature structure. Normal blood vessels have a hierarchical structure with uniformly running arteries, capillaries, and veins, whereas tumor blood vessels lose their hierarchy due to irregular branching and tortuous course, uneven vessel diameter, and uneven vessel density (Fig. 3). Capillaries also have immature structures, such as reduced vascular endothelial cell-to-cell junctions, insufficient pericyte coverage, and irregular basement membrane type IV collagen thickness. VEGF increases tumor vessel permeability by including vascular endothelial window structures and decreasing vascular endothelial cell adhesion via VE-cadherin internalization. As a result, blood vessel permeability is high in cancers with active angiogenesis. Furthermore, blood easily leaks out of the vessels, causing increased interstitial pressure. Tumor vessels with immature vessel walls are compressed in this environment, and blood circulation is poor. As a result, despite a dense vascular network, cancer tissues are frequently hypoxic [5]. Hypoxia promotes further immature angiogenesis in cancer tissues by inducing VEGF production via HIF-1, activation of cancer stem cells, malignant transformation of cancer via epithelial–mesenchymal transition (EMT) [5], and further enhancement of immature angiogenesis [6].

### Inhibitors of angiogenesis

The basic concept of starving primary tumors, as described above, gave rise to angiogenesis inhibitors, making vascular endothelial cells the second most important therapeutic target after cancer cells. Initially, it was assumed that, in contrast to cancer cells with genetic instability such as mutations, vascular endothelial cells, which are genetically stable, would be less susceptible to drug resistance [7]. Furthermore, tumor endothelial cells (TECs) are thought to support 50–100 cancer cells, implying that TEC-targeted drugs should have a high therapeutic efficacy. The benefits of these drugs include the fact that they are expected to have a high therapeutic effect with a low drug dosage and be much less toxic than traditional anticancer drugs. These are important critical context for the development of angiogenesis inhibitors.

Bevacizumab (Avastin), the first FDA-approved angiogenesis inhibitor, is a monoclonal antibody against human VEGF that was approved in 2004. Bevacizumab, which inhibits angiogenesis by inhibiting VEGF function, has improved the prognosis of many patients. Table 1 lists other angiogenesis inhibitors currently approved by the FDA.

**Table 1:** Characteristics of tumor vascular endothelial cells

Upregulation of angiocrine factor gene expression
Enhanced proliferation and migration
Decreased intracellular adhesion
High sensitivity to cytokines and growth factors
Stem-like phenotype
Chromosomal abnormality
Resistance (drug, nutrition depletion, and ROS)
Abnormal metabolism (enhanced glycolysis, and acid resistance)
High collagen synthesis ability
Heterogeneity

(1) Monoclonal anti-VEGF antibodies (bevacizumab); (2) small molecule tyrosine-kinase inhibitors (TKIs) (sorafenib, sunitinib, pazopanib, vandetanib, axitinib, and cabozantinib); (3) mammalian target of rapamycin (mTOR) inhibitors (sirolimus, everolimus, and temsirolimus).

As previously stated, bevacizumab, a monoclonal antibody against VEGF, is the most well-known VEGF antibody drug. Colorectal cancer, non-small cell lung cancer, ovarian cancer, cervical cancer, recurrent breast cancer, and malignant glioma are all treated with bevacizumab in Japan.

The number of VEGFR inhibitors (TKIs) is increasing as new drugs are developed. Sunitinib inhibits VEGFR and is approved in Japan for the treatment of renal cell carcinoma, imatinib-resistant gastrointestinal stromal tumor, and pancreatic neuroendocrine tumor. Sorafenib inhibits VEGFR1, VEGFR2, VEGFR3, PDGFR, c-KIT, FLT-3, RET, and RAF kinases, and is known to induce tumor cell apoptosis via MAPK signaling as well as angiogenesis. It is approved to treat advanced renal cancer, hepatocellular carcinoma, and thyroid cancer. Aflibercept is another angiogenesis inhibitor. A new angiogenesis inhibitor approved in Japan in 2017; it is a recombinant fusion protein of the VEGF-binding regions of VEGFR1 and VEGFR2 with the Fc portion of IgG antibodies [8]. It has a higher affinity for VEGF than anti-VEGF monoclonal antibodies.

Because the mTOR pathway is important in the PI3K/Akt signaling pathway and is required for angiogenesis, cell proliferation, and metabolism [9], mTOR inhibitors were developed. mTOR kinase inhibition inhibits downstream Akt signaling, inhibiting protein translation and cell proliferation. Because it regulates the expression of HIF-1, which is up-regulated in patients with von Hippel–Lindau gene deletion, mTOR inhibitors are approved for renal cell carcinoma [10, 11].

### Cancer immunity and angiogenesis inhibitors

The majority of current angiogenesis inhibitors target VEGF and its signaling pathway. As previously stated, VEGF signaling increases tumor blood vessel permeability, and blocking this signaling not only inhibits the proliferation but also improves blood flow by returning immature and permeable tumor blood vessels to their normal pericyte-surrounding stable structure (tumor vascular normalization). This is expected to improve the therapeutic effect of anticancer drugs taken concurrently [12]. Because cancer tissue is reoxygenated, vascular normalization is beneficial for radiation therapy.

Furthermore, tumor blood vessels are important in cancer immunity. CD8-positive cytotoxic lymphocytes (CTLs) that recognize cancer antigens must infiltrate into cancer tissues during the immune response induction process [13]. Moreover, CTLs must attack cancer cells via blood vessel transport to cancer tissues and migration between TECs to complete the cancer immune cycle. The tumor vasculature is crucial in cancer immunology. As a result, the role of tumor blood vessels in cancer immunity is also thought to be important. Angiogenesis inhibitors have recently been shown to increase the number of cells in cancer tissues and activate tumor immunity [14]. Furthermore, it has been demonstrated that VEGF inhibition activates tumor immunity. VEGF, for example, suppresses immunity by activating regulatory T cells (Treg), increasing the recruitment of immunosuppressive M2-type tumor-associated macrophages and bone marrow-derived suppressor cells, and inhibiting dendritic cell (DC) maturation. It has also been reported that VEGF/VEGFR signaling downregulates the expression of vascular endothelial cell adhesion factors VCAM and ICAM, inhibits CTL adhesion to vascular endothelium infiltrating cancer tissues, and induces CTL apoptosis via induction of FasL expression in vascular endothelial cells [15]. As a result, angiogenesis inhibitors are expected to inhibit the various immunosuppressive effects of VEGF while also promoting CTL mobilization by normalizing blood vessels. Combination therapy with anti-VEGF agents and immune checkpoint inhibitors is currently approved [16], and clinical trials for other types of cancer are ongoing, with the expectation that these combination therapies will improve efficacy.

### Tumor vascular endothelial cell characteristics

The mainstay of angiogenesis inhibitors, as described above, VEGF/VEGFR signaling inhibitors have improved the prognosis of many patients. However, as with many drugs, some obstacles have been identified that must be overcome.

Because VEGF is required for normal vascular endothelial cell survival, blocking VEGF signaling can damage normal blood vessels, and side effects (hypertension, proteinuria, bleeding, thrombosis, and gastrointestinal perforation) have been reported [17]. This is one of the reasons why the dosage of this medication should be kept to a minimum. Furthermore, the therapeutic effect is limited in some carcinomas. This is due to the fact that some cancers are less dependent on VEGF for angiogenesis, while others are not [18]. Drug resistance, which was not anticipated when angiogenesis inhibitors were being developed, has also been reported [19, 20]. This is thought to be due to the fact that cancer angiogenesis shifts to rely on angiogenesis-promoting factors other than VEGF during treatment.

Recent research has focused on the complex regulatory mechanisms of tumor angiogenesis and the diversity of tumor vascular endothelial cells. TECs differ from normal endothelial cells (NECs) in gene expression [21, 22], and TECs have also been found to have functional abnormalities. TECs have higher angiogenic activity than NECs [23]. Some TECs have stem cell characteristics [20], and vascular endothelial cells from prostate cancer cells differentiate into bone and cartilage, indicating their multilineage differentiation potential [24]. Furthermore, some populations express markers that are similar to cancer stem cells, such as ABCB1 and ALDH, which have been linked to drug resistance [19, 25, 26]. Moreover, TEC chromosomal aberrations have been reported [27, 28]. Chemotherapy has been shown to target drug-resistant vessels, and targeting drug-resistant vessels has been shown to improve the therapeutic effect [27, 28]. Tumor vascular endothelial cell characteristics should be considered in the treatment of angiogenesis inhibitor resistance.

Tumor vascular endothelial cells have also been proposed as a mechanism for their abnormality. In fact, it has been proposed that the presence of tissue stem cells in tissues serves as a source of endothelial cells during angiogenesis. The presence of vascular endothelial cells derived from cancer cells or cancer stem cells has been suggested in glioblastoma [29]. Although these findings are debatable, they do point to the diversity of tumor vascular endothelial cells.

The cancer microenvironment also influences tumor vascular endothelial cell diversity. Hypoxia, cytokine levels in cancer tissues, hypoxic environment, exosomes secreted by cancer cells, or miRNAs contained in them, for example, have been proposed to be responsible for various changes in TEC characteristics (e.g., gene expression, stemness, chromosomal aberrations, and drug resistance) [19, 30, 31].

The metabolic pathways of tumor vascular endothelial cells are also becoming clearer.

The Warburg effect demonstrates that cancer tissue metabolism is abnormal, but tumor vascular endothelial cells have different metabolic pathways than normal cells. Tumor vascular endothelial cells, like cancer cells, are dependent on the glycolytic regulator phosphofrucokinase-2/fructose-2, 6-bisphophatase 3 (PFKFB3) [32], and tumor vascular endothelial cells are resistant to the acidic environment of cancer tissue due to increased expression of carbonic anhydrase II (CAII) [33, 34].

It has also been reported that tumor vascular endothelial cells have unique mechanisms for the regulation of radical oxygen species produced in cancer tissues [34]. Furthermore, extracellular vesicle secreted from cancer, including VEGF, has been suggested as a novel mechanism for anti-VEGF antibody therapy [35].

Furthermore, single-cell RNA sequencing studies have revealed not only the diversity of vascular endothelial cells in normal tissues [36] but also the diversity of tumor vascular endothelial cells. Some tumor vascular endothelial cells in human lung cancer have also been shown to promote basement membrane degradation, immune cell migration, and collagen cross-linkage binding [37].

As a result, it is clear that different factors in the cancer microenvironment confer complex and diverse properties on vascular endothelial cells. It has been reported that tumor vascular endothelial cells secrete various factors that act on cells and stroma in the microenvironment to induce distant metastasis of cancer cells [38] and promote cancer fibrosis [39]. The development of a tumor vascular-selective therapeutic agent that targets tumor vascular endothelial cell characteristics may reduce the side effects of angiogenesis inhibitors (Table 1). Furthermore, ultrasound contrast agents that specifically bind to TEC ligands may be an appealing tool for cancer diagnosis.

### Future prospects of angiogenesis inhibitor therapy

Normalization of tumor blood vessels using angiogenesis inhibitors is critical, because it also leads to normalization of the microenvironment; however, hypoxia of tissues due to continued angiogenesis inhibition has been reported in some cancers [40, 41]. Because hypoxia can cause EMT and even malignant transformation of cancer, it has been proposed that angiogenesis inhibitors have a normalization window to prevent cancer tissue from becoming hypoxic again [42].

The significance of companion diagnosis in angiogenesis inhibitor therapy is also recognized to select the appropriate timing and duration of administration of the agent or the indicated patients. Apelin induced by hypoxia, for example, has been reported to be a marker of tumor vascular normalization and may be an indicator of vascular normalization [42].

Dr. Folkman proposed the utility of regulating angiogenesis in cancer therapy nearly 50 years ago, and research on angiogenesis has progressed dramatically since then, becoming now not only a basic medical research topic but also an important therapeutic target in clinical practice. Tumor angiogenesis is now known to be linked not only to the malignant transformation of cancer cells but also to tumor immunity and drug resistance. Understanding the various molecular characteristics of tumor vascular endothelial cells may lead to the development of new anti-angiogenic therapies in conjunction with companion diagnostics.

## References

[1] Folkman J. Tumor angiogenesis: therapeutic implications. N Engl J Med. 1971;285:1182–6. 4938153 10.1056/NEJM197111182852108

[2] Ferrara N. VEGF as a therapeutic target in cancer. Oncology. 2005;69:11–6. 16301831 10.1159/000088479

[3] Folkman J. Anti-angiogenesis: new concept for therapy of solid tumors. Ann Surg. 1972;175:409–16. 5077799 10.1097/00000658-197203000-00014 PMC1355186

[4] Keyt BA, Nguyen HV, Berleau LT, et al. Identification of vascular endothelial growth factor determinants for binding KDR and FLT-1 receptors Generation of receptor-selective VEGF variants by site-directed mutagenesis. J Biol Chem. 1996;271:5638–46. 8621427 10.1074/jbc.271.10.5638

[5] Martin JD, Fukumura D, Duda DG, et al. Reengineering the Tumor Microenvironment to Alleviate Hypoxia and Overcome Cancer Heterogeneity. Cold Spring Harb Perspect Med. 2016;6:a027094-a27125. 27663981 10.1101/cshperspect.a027094 PMC5131751

[6] Maishi N, Annan DA, Kikuchi H, et al. Tumor Endothelial Heterogeneity in Cancer Progression. Cancers (Basel). 2019;11:1511. 31600937 10.3390/cancers11101511 PMC6826555

[7] Kerbel RS. Inhibition of tumor angiogenesis as a strategy to circumvent acquired resistance to anti-cancer therapeutic agents. BioEssays. 1991;13:31–6. 1722975 10.1002/bies.950130106

[8] Ivy SP, Wick JY, Kaufman BM. An overview of small-molecule inhibitors of VEGFR signaling. Nat Rev Clin Oncol. 2009;6:569–79. 19736552 10.1038/nrclinonc.2009.130

[9] Gibbons JJ, Abraham RT, Yu K. Mammalian target of rapamycin: discovery of rapamycin reveals a signaling pathway important for normal and cancer cell growth. Semin Oncol. 2009;36:S3–17. 19963098 10.1053/j.seminoncol.2009.10.011

[10] Hudes GR. mTOR as a target for therapy of renal cancer. Clin Adv Hematol Oncol. 2007;5:772–4. 17998894

[11] Meric-Bernstam F, Mills GB. Mammalian target of rapamycin. Semin Oncol. 2004;31:10–7. 15799239 10.1053/j.seminoncol.2004.10.013

[12] Jain RK. Normalization of tumor vasculature: an emerging concept in antiangiogenic therapy. Science. 2005;307:58–62. 15637262 10.1126/science.1104819

[13] Chen DS, Mellman I. Oncology meets immunology: the cancer-immunity cycle. Immunity. 2013;39:1–10. 23890059 10.1016/j.immuni.2013.07.012

[14] Fukumura D, Kloepper J, Amoozgar Z, et al. Enhancing cancer immunotherapy using antiangiogenics: opportunities and challenges. Nat Rev Clin Oncol. 2018;15:325–40. 29508855 10.1038/nrclinonc.2018.29 PMC5921900

[15] Kim JM, Chen DS. Immune escape to PD-L1/PD-1 blockade: seven steps to success (or failure). Ann Oncol. 2016;27:1492–504. 27207108 10.1093/annonc/mdw217

[16] Huang Y, Goel S, Duda DG, et al. Vascular normalization as an emerging strategy to enhance cancer immunotherapy. Can Res. 2013;73:2943–8. 10.1158/0008-5472.CAN-12-4354 PMC3655127 23440426

[17] Gacche RN, Meshram RJ. Angiogenic factors as potential drug target: efficacy and limitations of anti-angiogenic therapy. Biochem Biophys Acta. 2014;1846:161–79. 24836679 10.1016/j.bbcan.2014.05.002

[18] Casanovas O, Hicklin DJ, Bergers G, et al. Drug resistance by evasion of antiangiogenic targeting of VEGF signaling in late-stage pancreatic islet tumors. Cancer Cell. 2005;8:299–309. 16226705 10.1016/j.ccr.2005.09.005

[19] Akiyama K, Ohga N, Hida Y, et al. Tumor endothelial cells acquire drug resistance by mdr1 up-regulation via vegf signaling in tumor microenvironment. Am J Pathol. 2012;180:1283–93. 22245726 10.1016/j.ajpath.2011.11.029

[20] Naito H, Kidoya H, Sakimoto S, et al. Identification and characterization of a resident vascular stem/progenitor cell population in preexisting blood vessels. EMBO J. 2011;31:1–14. 10.1038/emboj.2011.465 PMC3280559 22179698

[21] Hida K, Maishi N, Annan DA, et al. Contribution of tumor endothelial cells in cancer progression. Int J Mol Sci. 2018;19:223. 29695087 10.3390/ijms19051272 PMC5983794

[22] Annan DA, Kikuchi H, Maishi N, et al. Tumor endothelial cell-a biological tool for translational cancer research. Int J Mol Sci. 2020;21:332. 32375250 10.3390/ijms21093238 PMC7247330

[23] Matsuda K, Ohga N, Hida Y, et al. Isolated tumor endothelial cells maintain specific character during long-term culture. Biochem Biophys Res Commun. 2010;394:947–54. 20302845 10.1016/j.bbrc.2010.03.089

[24] Dudley AC, Khan ZA, Shih SC, et al. Calcification of multipotent prostate tumor endothelium. Cancer Cell. 2008;14:201–11. 18772110 10.1016/j.ccr.2008.06.017 PMC2604136

[25] Ohmura-Kakutani H, Akiyama K, Maishi N, et al. Identification of Tumor Endothelial Cells with High Aldehyde Dehydrogenase Activity and a Highly Angiogenic Phenotype. PLoS ONE. 2014;9:e113910–7. 25437864 10.1371/journal.pone.0113910 PMC4250080

[26] Naito H, Wakabayashi T, Kidoya H, et al. Endothelial Side Population Cells Contribute to Tumor Angiogenesis and Antiangiogenic Drug Resistance. Can Res. 2016;76:3200–10. 10.1158/0008-5472.CAN-15-2998 27197162

[27] Hida K, Hida Y, Amin DN, et al. Tumor-associated endothelial cells with cytogenetic abnormalities. Cancer Res. 2004;64:8249–55. 15548691 10.1158/0008-5472.CAN-04-1567

[28] Akino T, Hida Y, et al. Cytogenetic abnormalities of tumor-associated endothelial cells in human malignant tumors. Am J Pathol. 2010;175:2657–67. 10.2353/ajpath.2009.090202 PMC2789618 19875502

[29] Ricci-Vitiani L, Pallini R, Biffoni M, et al. Tumour vascularization via endothelial differentiation of glioblastoma stem-like cells. Nature. 2010;468:1–7. 10.1038/nature09557 21102434

[30] Tominaga N, Kosaka N, Ono M, et al. Brain metastatic cancer cells release microRNA-181c-containing extracellular vesicles capable of destructing blood-brain barrier. Nat Commun. 2015;6:6716. 25828099 10.1038/ncomms7716 PMC4396394

[31] Annan DA, Maishi N, Soga T, et al. Carbonic anhydrase 2 (CAII) supports tumor blood endothelial cell survival under lactic acidosis in the tumor microenvironment. Cell Commun Signal. 2019;17:169. 31847904 10.1186/s12964-019-0478-4 PMC6918655

[32] De Bock K, Georgiadou M, Schoors S, et al. Role of PFKFB3-driven glycolysis in vessel sprouting. Cell. 2013;154:651–63. 23911327 10.1016/j.cell.2013.06.037

[33] Okuno Y, Nakamura-Ishizu A, Otsu K, et al. Pathological neoangiogenesis depends on oxidative stress regulation by ATM. Nat Med. 2012;18:1208–16. 22797809 10.1038/nm.2846

[34] Hojo T, Maishi N, Towfik AM, et al. ROS enhance angiogenic properties via regulation of NRF2 in tumor endothelial cells. Oncotarget. 2017;8:45484–95. 28525375 10.18632/oncotarget.17567 PMC5542202

[35] Ma S, Mangala LS, Hu W, et al. CD63-mediated cloaking of VEGF in small extracellular vesicles contributes to anti-VEGF therapy resistance. Cell Rep. 2021;36:109549. 34407412 10.1016/j.celrep.2021.109549 PMC8422976

[36] Kalucka J, de Rooij L, Goveia J, et al. Single-Cell Transcriptome Atlas of Murine Endothelial Cells. Cell. 2020;180:e20. 10.1016/j.cell.2020.01.015 32059779

[37] Goveia J, Rohlenova K, Taverna F, et al. An Integrated Gene Expression Landscape Profiling Approach to Identify Lung Tumor Endothelial Cell Heterogeneity and Angiogenic Candidates. Cancer Cell. 2020;37:13. 10.1016/j.ccell.2020.03.002 32183954

[38] Maishi N, Ohba Y, Akiyama K, et al. Tumour endothelial cells in high metastatic tumours promote metastasis via epigenetic dysregulation of biglycan. Sci Rep. 2016;6:1–13. 27295191 10.1038/srep28039 PMC4904795

[39] Cong L, Maishi N, Annan DA, et al. Inhibition of stromal biglycan promotes normalization of the tumor microenvironment and enhances chemotherapeutic efficacy. Breast Cancer Res. 2021;23:51. 33966638 10.1186/s13058-021-01423-w PMC8108358

[40] Ebos JML, Lee CR, Cruz-Munoz W, et al. Accelerated metastasis after short-term treatment with a potent inhibitor of tumor angiogenesis. Cancer Cell. 2009;15:232–9. 19249681 10.1016/j.ccr.2009.01.021 PMC4540346

[41] Sato M, Maishi N, Hida Y, et al. Angiogenic inhibitor pre-administration improves the therapeutic effects of immunotherapy. Cancer Med. 2023. 10.1002/cam4.5696. 36808261 10.1002/cam4.5696 PMC10166916

[42] Zhang L, Takara K, Yamakawa D, et al. Apelin as a marker for monitoring the tumor vessel normalization window during antiangiogenic therapy. Cancer Sci. 2015;107:36–44. 26475217 10.1111/cas.12836 PMC4724822

