# Correction: Beyond starving cancer: anti-angiogenic therapy

**DOI:** 10.1007/s10396-024-01490-4

**Published:** 2024-08-19

**Authors:** Kyoko Hida, Nako Maishi, Aya Matsuda, Li Yu

**Affiliations:** Vascular Biology and Molecular Pathology, Faculty and Graduate School of Dental Medicine, Hokkaido University, N13 W7 Kita-Ku, Sapporo, 060-8586 Japan

**Correction: Journal of Medical Ultrasonics** 10.1007/s10396-023-01310-1

The article Beyond starving cancer: anti‑angiogenic therapy, written by Kyoko Hida, Nako Maishi, Aya Matsuda and Li Yu, was originally published electronically on the publisher’s internet portal on 28 May 2023 without open access. With the society’s decision to opt for Open Choice the copyright of the article changed on 2 August 2024 to © The Author(s) 2023 and the article is forthwith distributed under a Creative Commons Attribution 4.0 International License, which permits use, sharing, adaptation, distribution and reproduction in any medium or format, as long as you give appropriate credit to the original author(s) and the source, provide a link to the Creative Commons licence, and indicate if changes were made. The images or other third party material in this article are included in the article’s Creative Commons licence, unless indicated otherwise in a credit line to the material. If material is not included in the article’s Creative Commons licence and your intended use is not permitted by statutory regulation or exceeds the permitted use, you will need to obtain permission directly from the copyright holder. To view a copy of this licence, visit http://creativecommons.org/licenses/by/4.0.

